# Screening for inherited metabolic disorders in patients with Familial Mediterranean Fever

**DOI:** 10.1186/1546-0096-13-S1-P97

**Published:** 2015-09-28

**Authors:** E Kiykim, AC Aktuglu-Zeybek, K Barut, T Zubarioglu, MS Cansever, A Aydin, O Kasapcopur

**Affiliations:** 1 Istanbul University, Cerrahpasa Medical Faculty, Pediatric Metabolic Diseases, Istanbul, Turkey; 2 Istanbul University, Cerrahpasa Medical Faculty, Pediatric Rheumatology, Istanbul, Turkey

## Introduction

Familial Mediterranean fever (FMF) is an autosomal recessive auto-inflammatory disease, presenting with recurrent episodes of fever and polyserositis. Diagnosis of FMF is may be challenging especially in pediatric population. Mitochondrial fatty acid oxidation disorders and porphyrias can present with periodic abdominal and muscle pain. Incidence of both FMF and inherited metabolic disorders (IMD) are increased in Turkish patients partially due to high consanguinity rates.

## Objectives

The aim of the present study is determine the inherited metabolic disorders in differential diagnosis of Turkish pediatric FMF patients.

## Methods

174 patients who were diagnosed as FMF enrolled the study. In all patients, a fasting dry spot blood sample was taken for acyl-carnitine analyses by tandem mass spectrometry. Fresh, light-protected spot urine test was performed for porphobilinogen screening. Second-tier test with urine organic acid analysis and urine porphyrin metabolites were performed if pathologic findings were detected in acyl-carnitine profile or in porphobilinogen screening, for confirmation. An age matched healthy 50 children served as control group.

## Results

Of the 174 patients diagnosed with FMF, none of our patients was diagnosed with porphyria; two patients with fatty acid oxidation defect, one with multiple acyl-CoA dehydrogenase deficiency and one with possible medium-chain acyl-CoA dehydrogenase deficiency were detected during the study.

## Conclusion

Our data revealed that screening for porphobilinogen for pediatric FMF patients is unnecessary, but an investigation of tandem mass based acyl-carnitine analyses can be helpful for the differential or additional diagnosis of FMF in developing countries that does not have nationwide expanded newborn screening programme.

